# Correction: Wiedermann et al. Health Information Use and Trust: The Role of Health Literacy and Patient Activation in a Multilingual European Region. *Int. J. Environ. Res. Public Health* 2025, *22*, 570

**DOI:** 10.3390/ijerph22081165

**Published:** 2025-07-23

**Authors:** Christian J. Wiedermann, Verena Barbieri, Stefano Lombardo, Timon Gärtner, Patrick Rina, Klaus Eisendle, Giuliano Piccoliori, Adolf Engl, Dietmar Ausserhofer

**Affiliations:** 1 Institute of General Practice and Public Health, Claudiana College of Health Professions, 39100 Bolzano, Italygiuliano.piccoliori@am-mg.claudiana.bz.it (G.P.);; 2 Provincial Institute for Statistics of the Autonomous Province of Bolzano, South Tyrol (ASTAT), 39100 Bolzano, Italy; stefano.lombardo@provincia.bz.it (S.L.);; 3 Directorate, Claudiana College of Health Professions, 39100 Bolzano, Italy; 4 Claudiana Research, Claudiana College of Health Professions, 39100 Bolzano, Italy

In the original publication [1], there was a mistake in Table 1 as published. The proportions of health literacy levels (Inadequate, Problematic, Adequate) were calculated using the total sample size of 2090 as the denominator, inadvertently including 441 participants with missing health literacy data. The corrected proportions are based on valid cases only. The corrected Table 1 appears below. The authors state that the scientific conclusions are unaffected. This correction has been approved by the Academic Editor. The original publication has also been updated.

**Table 1:** Demographic and other characteristics of the sample were the weighted distribution of categorical variables, including sociodemographic characteristics, health literacy (HLS-EU-Q16), patient activation (PAM-10), and self-reported health status (*n* = 2090).

Variable	Category	Weighted *n*	Weighted Proportion %	95% CI Low %	95% CI High %
Gender	Female	1158	55.5	54.2	56.8
	Male	932	44.5	43.2	45.8
Age Group (Years) ^1^	18–34	378	18.1	16.9	19.3
	35–54	643	30.7	29.3	32.1
	55+	1070	51.2	49.7	52.7
Native Language	German	1395	66.8	65.5	68.1
	Italian	499	23.9	22.7	25.1
	Other	194	9.3	8.5	10.1
Citizenship	Italian	2009	96.1	95.4	96.8
	Other	81	3.9	3.2	4.6
Community Type	Urban	387	18.5	17.3	19.7
	Rural	1703	81.5	80.3	82.7
Living Situation	Alone	378	18.1	17.0	19.3
	With partner/family	1327	63.5	62.1	64.9
	With children	787	37.7	36.3	39.1
Educational Level	Middle school or lower	487	23.3	22.0	24.6
	Vocational school	670	32.1	30.7	33.5
	High school	533	25.6	24.3	26.9
	University	396	19.0	17.9	20.1
Health Literacy ^2^	Inadequate	266	16.1	14.2	18.0
	Problematic	559	33.9	31.5	36.3
	Adequate	824	50.0	47.4	52.5
PAM-10 ^3^	Disengaged	340	16.3	15.3	17.3
	Becoming aware	888	42.6	41.2	44.0
	Taking action	648	31.1	29.8	32.4
	Maintaining	204	9.8	9.0	10.6
Health Status(0–100) ^4^	Poor (0–50)	255	12.2	11.3	13.1
	Fair (51–75)	625	29.8	28.5	31.1
	Good (76–90)	820	39.1	37.8	40.4
	Excellent (91–100)	390	18.9	17.9	19.9

^1^ weighted mean age of 53.8 years (standard deviation of 17.6); median of 54 years (interquartile range of 39–69). ^2^ HLS-EU-Q16: health literacy was grouped into inadequate (0–25), problematic (26–33), and adequate (34–50). ^3^ PAM-10: patient activation was categorised into four levels: low (≤47.0, disengaged) to high (≥72.5, maintaining behaviours). ^4^ Health status: categorised into poor (0–50), fair (51–75), good (76–90), and excellent (91–100). CI, confidence interval.
