# Supplementary material for: Health Information Use and Trust: The Role of Health Literacy and Patient Activation in a Multilingual European Region
Source: Int J Environ Res Public Health. 2025 Apr 5;22(4):570. doi: 10.3390/ijerph22040570 (PMC12027244; doi:10.3390/ijerph22040570)
Supplement: Supplementary file 1 [file ijerph-22-00570-s001.zip › ijerph-3510640 File S1.pdf]

Dear Sir or Madam, Thank you for your participation in this study conducted by ASTAT in collaboration with the Institute of General Practice and Public Health (Claudiana)

You will need about 10 minutes to complete the questionnaire.

The anonymised data is collected exclusively for research purposes.

The information collected in this survey is protected by statistical confidentiality (art. 9 of Legislative Decree no. 322/1989) and is subject to legislation on the protection of personal data (EU Regulation no. 679/2016 and Legislative Decree no. 196/2003, as last amended by Legislative Decree no. 101 of 10 August 2018).

Part A: SOCIODEMOGRAPHICAL DATA

A1. Gender

Female ☐

Male ☐

A2. Age (in completed years)

☐☐☐

LimeSurvey

A3. Municipality of residence

Aldein ☐
Andrian ☐
Altrei ☐
Eppan a.d.Weinstr. ☐
Hafling ☐
Abbey ☐
Barbian ☐
Bolzano ☐
Prague ☐
Burner ☐
Bressanone ☐
Branzoll ☐
Bruneck ☐
Kuens ☐
Kaltern a.d.Weinstr. ☐
Free field ☐
Sand in Taufers ☐
Castelbello-Ciardes ☐
Castelrotto ☐
Tscherms ☐
Chienes ☐
Klausen ☐
Carneid ☐
Kurtatsch a.d.Weinstr. ☐
Kurtinig a.d.Weinstr. ☐
Corvara ☐
Graun in the Vinschgau Valley ☐
Dobbiaco ☐
New market ☐
Palatinate ☐
Fiè allo Sciliar ☐

A4. Completed training

Secondary school leaving certificate or [ ] er vocational

school leaving certificate (2-3 years) [ ]

Matura [ ]

University degree (and higher) [ ]

A5. Citizenship

Italian [ ]

Foreign [ ]

A6. Mother tongue

German [ ]

Italian Ladin [ ]

Other language [ ]

More than one [ ]

A7. Do you live with the following people all the time?

alone [ ]

with partner [ ]

with my children (at least one) [ ]

with my parents [ ]

with other family members [ ]

A8. Do you work in the health and social services sector?

Y [ ]

es [ ]

No

Part B: LIVING HABITS

B1. How often do you engage in the following behaviours?

	never	rarely	often	always
I exercise to stay fit I make	☐	☐	☐	☐
sure I have a balanced diet	☐	☐	☐	☐
I take vitamins I go to	☐	☐	☐	☐
the dentist for regular check-ups	☐	☐	☐	☐
I control my weight I limit my	☐	☐	☐	☐
consumption of coffee, sugar and fat	☐	☐	☐	☐
I collect information about things that affect my health I look out for	☐	☐	☐	☐
possible signs of serious health problems I take health supplements	☐	☐	☐	☐
I go to the doctor regularly for check-ups	☐	☐	☐	☐
I use dental floss I	☐	☐	☐	☐
discuss health issues with friends, neighbours, relatives	☐	☐	☐	☐
I avoid smoking I brush	☐	☐	☐	☐
my teeth I get	☐	☐	☐	☐
immunised I get	☐	☐	☐	☐
enough sleep	☐	☐	☐	☐
	☐	☐	☐	☐

LimeSurvey

Part C: HEALTH LITERACY

C1. your opinion, how easy is it...

	Very simple	fairly simple	quite difficult	very difficult	don't know
...information about therapies for diseases that you to find?	☐	☐	☐	☐	☐
...to find out where you can get professional help if you are ill?	☐	☐	☐	☐	☐
...to understand what your doctor is telling you?	☐	☐	☐	☐	☐
...understand your doctor's or pharmacist's instructions on how to take your prescribed medication?	☐	☐	☐	☐	☐
...to judge when you need a second opinion from another person. should consult a doctor?	☐	☐	☐	☐	☐
...to decisions about your illness with the help of the information your doctor gives you?	☐	☐	☐	☐	☐
...to follow the instructions of your doctor or pharmacist?	☐	☐	☐	☐	☐
...to find information about support options for mental health problems such as stress or depression?	☐	☐	☐	☐	☐
...understand health warnings about behaviours such as smoking, lack of exercise or excessive drinking?	☐	☐	☐	☐	☐
...to understand why you need check-ups? (cancer screening, blood sugar test, blood pressure)	☐	☐	☐	☐	☐
...to judge whether the information about health risks in the media is trustworthy? (Television, Internet, ...)	☐	☐	☐	☐	☐
...to decide how protect yourself from diseases based on information from the media?	☐	☐	☐	☐	☐
...find information about behaviours that good for your mental well-being? (Meditation, physical Exercise, walking, Pilates etc.)	☐	☐	☐	☐	☐
...health advice from family members or friends to understand?	☐	☐	☐	☐	☐
...understand information in the media about how you can improve your health?	☐	☐	☐	☐	☐
...assess which everyday habits are related to your health? (drinking and eating habits, exercise, etc.)	☐	☐	☐	☐	☐

Part D: HEALTH: Your situation TODAY

D1. Your current state of health: from 0 (the worst possible state of health) to 100 (the best)

| | | | | | | | | | |

D2. Do you suffer from or have you been diagnosed with the following chronic diseases?

Lung diseases (e.g. asthma, chronic obstructive pulmonary disease) ☐

Cardiovascular diseases (e.g. coronary heart disease, atrial fibrillation, heart failure, Stroke/circulatory disorder of the brain) ☐

High blood pressure ☐

Kidney diseases ☐

Diseases of the immune system ☐

Tumour diseases ☐

Metabolic diseases (e.g. diabetes, obesity) ☐

Liver diseases ☐

Mental illness, depression and/or anxiety disorder ☐

none of these ☐

D3. How often in the last 12 months have you been to the ....?

General practitioner

First aid

Specialist

Hospital

Part E: ACTIVATION

E1. How much do you agree with the following statements?

	do not agree at all	do not agree	agree	fully agreed	does not concern me
Ultimately, I am the one who is responsible for my health	☐	☐	☐	☐	☐
The most important thing for my health is to take an active role in my healthcare	☐	☐	☐	☐	☐
I know the effects of all the medication I have been prescribed become	☐	☐	☐	☐	☐
I am confident that I know when I need to go to the doctor and when I can treat a health problem myself	☐	☐	☐	☐	☐
I am convinced that I can tell my GP about my concerns, even if he doesn't talk to me about them	☐	☐	☐	☐	☐
I am convinced that I can carry out medical treatment myself at home if necessary	☐	☐	☐	☐	☐
I have so far been to make changes to my lifestyle habits - such as healthy eating and exercise - and maintain	☐	☐	☐	☐	☐
I know how to prevent health problems	☐	☐	☐	☐	☐
I am convinced that we will find solutions when new health issues arise. Problems arise	☐	☐	☐	☐	☐
I am convinced that I can continue to make changes to my lifestyle habits - such as healthy eating and physical exercise - even in stressful times	☐	☐	☐	☐	☐

Part F: INFORMATION SOURCES

F1. How do you generally find out about health issues?

	regularly	occasionally	rarely	never
Articles in newspapers or magazines	☐	☐	☐	☐
Television or radio programmes on health topics	☐	☐	☐	☐
Conversations with friends or acquaintances	☐	☐	☐	☐
Discussions with specialists, e.g. doctors or nursing staff	☐	☐	☐	☐
Events or courses	☐	☐	☐	☐
Specialist literature, e.g. health encyclopaedias or how-to books	☐	☐	☐	☐
By chance on the Internet, e.g. while surfing	☐	☐	☐	☐
On the Internet, through a targeted search	☐	☐	☐	☐
in Internet forums in which personal questions are asked or answered become	☐	☐	☐	☐
on social networks (Facebook, Instagram, ...)	☐	☐	☐	☐

F2. In general, how much do you trust ... in health matters? ?

	very	quite	little	not at all
Your family doctor	☐	☐	☐	☐
the specialists in the outpatient clinics or hospitals	☐	☐	☐	☐
the pharmacists	☐	☐	☐	☐
the advice of friends or relatives	☐	☐	☐	☐
Information from books	☐	☐	☐	☐
Information from the Internet	☐	☐	☐	☐
Your own feeling or experience	☐	☐	☐	☐
the nurses	☐	☐	☐	☐

F3. **On which health-related topics would you like to see more information in South Tyrol?**

Part G: SLEEP QUALITY

G1. **What time did you usually go to bed at night last month?**

- 20.00 ☐
- 20.30 ☐
- 21.00 ☐
- 21.30 ☐
- 22.00 ☐
- 22.30 ☐
- 23.00 ☐
- 23.30 ☐
- 24.00 ☐
- after midnight ☐

G2. **In the last month, how long did it usually take you to fall asleep at night? (in minutes)**

G3. What time did you usually get up in the morning last month?

- [ ] 5.00 or earlier
- [ ] 5.30
- [ ] 6.00
- [ ] 6.30
- [ ] 7.00
- [ ] 7.30
- [ ] 8.00
- [ ] 8.30
- [ ] 9.00
- [ ] 9.30 or later

G4. How many hours did you actually sleep per night last month? (can be less than the hours spent in bed)

G5. How often in the last month have you woken up in the middle of the night or early morning and been unable to go back to sleep?

- [ ] never in the last month
- [ ] Less than once a week
- [ ] 1 or 2 times a week
- [ ] 3 times a week or more

G6. How would you rate the overall quality of your sleep in the last month?

- [ ] very good
- [ ] pretty good
- [ ] pretty bad
- [ ] very bad

G7. In the last month, how often have you taken medication (prescribed by a doctor or over-the-counter) to help you sleep?

- never in the last month ☐
- Less than once a week ☐
- 1 or 2 times a week ☐
- 3 times a week or more ☐

Thank you very much! The results of the survey will be published on the ASTAT website in a few months' time.
