# Supplementary material for: Health Information Use and Trust: The Role of Health Literacy and Patient Activation in a Multilingual European Region
Source: Int J Environ Res Public Health. 2025 Apr 5;22(4):570. doi: 10.3390/ijerph22040570 (PMC12027244; doi:10.3390/ijerph22040570)
Supplement: Supplementary file 1 [file ijerph-22-00570-s001.zip › ijerph-3510640-supplementary.pdf]

Supplementary Materials

1. Overview

This document provides additional analyses and tables that complement the findings presented in the main manuscript. The supplementary materials include detailed subgroup analyses on health information source use and trust, as well as graphical representations of response distributions.

2. Additional Analyses

2.1. Sociodemographic Differences in the Use of Health Information Sources

Table S1 presents the distribution of health information source utilization by linguistic group. German speakers were more likely to use newspapers, magazines, and healthcare professionals, while Italian speakers engaged more with social media. Non-German speakers showed a slight preference for targeted internet searches. Although statistically significant, these differences had small effect sizes.

Table S1: Health information source utilization by linguistic group.

Health Information Source	Linguistic Group	Low Frequency [1]		High Frequency [1]	
		Count	Percentage	Count	Percentage
Newspapers/Magazines	German	644	46.0	754	53.9
	Italian	260	52.1	239	47.9
	Other	65	33.7	128	66.3
TV/Radio	German	678	48.5	720	51.5
	Italian	243	48.7	256	51.3
	Other	80	41.4	113	58.5
Friends/Acquaintances	German	877	62.7	521	37.3
	Italian	329	65.9	170	34.1
	Other	114	59.1	79	40.9
Healthcare Professionals	German	777	55.6	621	44.4
	Italian	321	64.3	178	35.7
	Other	100	51.8	93	48.2
Events/Courses	German	244	17.4	1154	82.5
	Italian	103	20.6	396	79.4
	Other	45	23.3	148	76.7
Specialist Literature	German	399	28.5	999	71.5
	Italian	125	25.0	374	75.0
	Other	55	28.5	138	71.5
Incidental Internet Use	German	561	40.1	837	59.9
	Italian	231	46.3	268	53.7
	Other	94	48.7	99	51.3
Targeted Internet Search	German	750	53.6	648	46.4
	Italian	298	59.7	201	40.3
	Other	122	63.2	71	36.8
Internet Forums	German	267	19.1	1131	80.9
	Italian	115	23.0	384	77.0
	Other	53	27.5	140	72.5

Social Media	German	291	20.8	1107	79.2
	Italian	136	27.2	363	72.7
	Other	60	31.1	133	68.9

[1] Low frequency includes participants who reported using the source 'seldom' or 'never,' while high frequency includes those who reported using it 'regularly' or 'sometimes.' Data are presented as absolute counts and percentages within each linguistic group.

Table S2 provides a detailed breakdown of the statistical results for these associations, including Chi-Squared values (χ^2), degrees of freedom (df), p-values, and effect sizes (Cramér's V). It highlights the strongest gender-based differences in reliance on friends/acquaintances for health information, as well as education-related differences in digital health information-seeking behavior.

Table S2: Association between sociodemographic factors and health information sources.

**Sociodemographic Factor**	**Information Source**	**Chi Squared χ^2**	**df**	**p-value**	**Cramér's V**	**Effect Size [1]**
Gender (Male/Female)	Newspapers/Magazines	46.166	3	< 0.001	0.149	Small
	TV/Radio	31.946	3	< 0.001	0.124	Small
	Friends/Acquaintances	75.245	3	< 0.001	0.190	Small
	Healthcare Professionals	16.898	3	< 0.001	0.090	Small
	Events/Courses	53.483	3	< 0.001	0.160	Small
	Specialist Literature	40.455	3	< 0.001	0.139	Small
	Incidental Internet Use	19.798	3	0.013	0.072	Small
	Targeted Internet Search	22.180	3	< 0.001	0.103	Small
	Internet Forums	5.910	3	0.116	0.053	Very small
	Social Media	28.334	3	< 0.001	0.116	Small
**Education Level** (Low, Medium, High) [2]	Newspapers/Magazines	27.997	6	< 0.001	0.082	Small
	TV/Radio	11.935	6	0.063	0.053	Very small
	Friends/Acquaintances	21.779	6	0.001	0.072	Small
	Healthcare Professionals	73.350	6	< 0.001	0.132	Small
	Events/Courses	106.148	6	< 0.001	0.159	Small
	Specialist Literature	85.430	6	< 0.001	0.143	Small
	Incidental Internet Use	96.281	6	< 0.001	0.151	Small
	Targeted Internet Search	209.683	6	< 0.001	0.224	Moderate
	Internet Forums	58.874	6	< 0.001	0.119	Small
	Social Media	40.336	6	< 0.001	0.098	Small
**Linguistic Group** (Italian, German, Other)	Newspapers/Magazines	35.671	6	< 0.001	0.092	Small
	TV/Radio	6.214	6	0.394	0.038	Very small...
	Friends/Acquaintances	6.7234	6	0.347	0.040	Small
	Healthcare Professionals	28.028	6	< 0.001	0.082	Small
	Events/Courses	20.084	6	0.003	0.069	Very small
	Specialist Literature	15.145	6	0.019	0.060	Very small
	Incidental Internet Use	32.6178	6	< 0.001	0.088	Small
	Targeted Internet Search	28.636	6	< 0.001	0.083	Small
	Internet Forums	15.076	6	0.0120	0.060	Very small

	Social Media	47.263	6	< 0.001	0.106	Small

[1] Cramér's effect size guide: Very Small = ≤ 0.07; Small = 0.07–0.2; Moderate = 0.2–0.3; Large = ≥ 0.3.
[2] Education: Lower (middle school or vocational), medium (high school), high (university).

2.2. Sociodemographic Differences in Trust in Health Information Sources

Table S3 details trust in health information sources by education level. Trust in healthcare professionals was consistently high across all education levels, while trust in the internet and family members showed greater variation. Lower education levels correlated with higher reliance on family advice, whereas higher education levels were associated with greater trust in books and professional sources.

Table S3: Trust in health information source by education level group.

Trust Source	Education Level	Low Trust		High Trust	
		Count	Percentage	Count	Percentage
Your GP	Low	85	10.4	735	89.6
	Middle	48	9.1	480	90.9
	High	23	8.5	248	91.5
Private/Public Specialists	Low	76	9.5	744	90.5
	Middle	50	9.5	478	90.5
	High	21	7.7	250	92.3
Pharmacist	Low	122	15.1	698	84.9
	Middle	69	13.1	459	86.9
	High	31	11.4	240	88.6
Family–Parents	Low	520	64.5	286	35.5
	Middle	199	37.7	329	62.3
	High	76	28.1	195	71.9
Books	Low	183	22.7	623	77.3
	Middle	94	17.8	434	82.2
	High	40	14.7	231	85.3
Internet	Low	610	76.3	189	23.7
	Middle	280	53.1	248	46.9
	High	105	38.8	166	61.2
Own Feelings	Low	89	11.1	710	88.9
	Middle	59	11.2	469	88.8
	High	26	9.6	245	90.4
Nurses	Low	149	18.6	650	81.4
	Middle	91	17.2	437	82.8
	High	38	14.0	233	86.0

[1] Low trust includes participants who reported trusting the source 'a little' or 'not at all,' while high trust includes those who reported trusting it 'very much' or 'rather.' Data are presented as absolute counts and percentages within each education level (Low, Middle, High).

2.3. Age Differences in the Use and Trust of Health Information Sources

Tables S3 summarize age-related differences in health information-seeking behavior. Older individuals predominantly used traditional sources like newspapers and television, while younger individuals relied more on digital sources, particularly targeted internet searches and social media. Trust in professional sources was stable across age groups, though younger individuals exhibited greater trust in the internet.

Table S4: Age differences in the use and trust of health information sources.

Variable	ANOVA				Effect Size [1]
	F	df	p-value	ω^2	
Source					
Targeted Internet Search	144.76	(3, 2086)	< 0.001	0.171	Large
Social Media	95.86	(3, 2086)	< 0.001	0.120	Moderate
Incidental Internet Use	88.67	(3, 2086)	< 0.001	0.112	Moderate
Internet Forums	48.91	(3, 2086)	< 0.001	0.064	Moderate
TV/Radio	38.24	(3, 2086)	< 0.001	0.051	Small
Newspapers/Magazines	22.43	(3, 2086)	< 0.001	0.030	Small
Friends/Acquaintances	16.67	(3, 2086)	< 0.001	0.022	Small
Events/Courses	15.12	(3, 2086)	< 0.001	0.020	Small
Healthcare Professionals	2.60	(3, 2086)	0.050	0.002	Small
Specialist Literature	1.72	(3, 2086)	0.162	0.001	Very small
Trust					
Internet	88.723	(3, 2086)	< .001	0.112	Moderate
Books	40.963	(3, 2086)	< .001	0.054	Small
Family-Parents	9.953	(3, 2086)	< .001	0.013	Small
Your GP	7.115	(3, 2086)	< .001	0.009	Small
Private/Public Specialists	5.037	(3, 2086)	0.002	0.006	Small
Nurses	1.516	(3, 2086)	0.209	0.0007	Very small
Own Feelings	1.409	(3, 2086)	0.238	0.0006	Very small
Pharmacist	1.314	(3, 2086)	0.268	0.0005	Very small

[1] Effect size interpretation: Very Small (≤ 0.01), Small (0.01 – 0.06), Moderate (0.06 – 0.14), and Large (≥ 0.14) [11].

Table S4 provides the corresponding statistical analysis for these trust differences, including Chi-Squared tests and effect sizes.

Table S5: Association between sociodemographic factors and trust in health information sources.

Sociodemographic Factor	Trust	Chi Squared			Cramér's V	Effect Size [1]
		χ^2	df	*p*-value		
Gender (Male/Female)	Your GP	0.751	3	0.861	0.019	Very small
	Private/Public Specialists	0.495	3	0.920	0.015	Very small
	Pharmacist	11.547	3	0.009	0.074	Small
	Family-Parents	5.113	3	0.146	0.049	Very small
	Books	10.689	3	0.014	0.072	Small
	Internet	1.720	3	0.632	0.028	Very small

	Own Feelings	7.931	3	0.047	0.062	Very small
	Nurses	5.807	3	0.121	0.052	Very small
Education Level (Low, Medium, High) [2]	Your GP	9.764	6	0.135	0.068	Very small
	Private/Public Specialists	14.582	6	0.024	0.084	Small
	Pharmacist	18.721	6	0.005	0.096	Small
	Family-Parents	32.409	6	< 0.001	0.126	Small
	Books	21.376	6	0.002	0.101	Small
	Internet	27.814	6	< 0.001	0.115	Small
	Own Feelings	16.295	6	0.012	0.089	Small
	Nurses	12.708	6	0.049	0.078	Small
Linguistic Group (Italian, German, Other)	Your GP	5.217	6	0.516	0.050	Very small
	Private/Public Specialists	18.013	6	0.006	0.093	Small
	Pharmacist	21.567	6	0.001	0.101	Small
	Family-Parents	57.912	6	< 0.001	0.166	Small
	Books	7.857	6	0.249	0.061	Very small
	Internet	13.161	6	0.041	0.079	Small
	Own Feelings	23.130	6	< 0.001	0.107	Small
	Nurses	9.060	6	0.170	0.066	Very Small

[1] Cramér's effect size guide: Very Small = ≤ 0.07; Small = 0.07–0.2; Moderate = 0.2–0.3; Large = ≥ 0.3.

[2] Education: Lower (middle school or vocational), medium (high school), high (university).

3. Supplementary Figures

Figure S1 shows the frequency distribution of responses regarding health information sources. Traditional media were commonly referenced but not primary sources. Digital sources, including internet searches, were widely used, whereas internet forums and social media were less frequently consulted. Friends and acquaintances were common sources, while healthcare professionals were consulted less often. Specialist literature and educational events were the least frequently used.

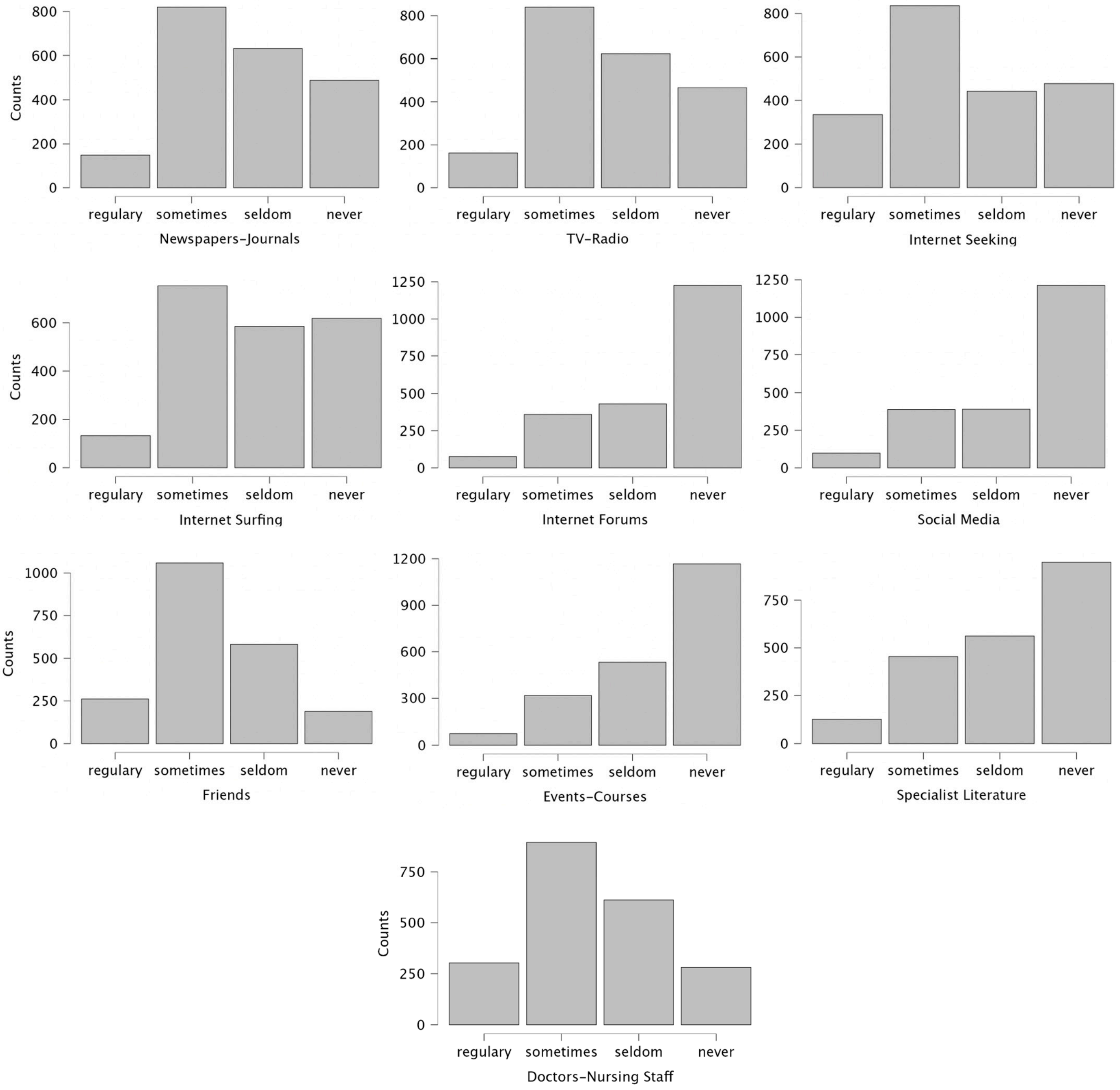

Figure S1: Distribution of trust ratings for various sources of health information (n = 2090).

Figure S2 illustrates trust ratings for different health information sources. Healthcare professionals, including general practitioners and specialists, received the highest trust levels, followed by pharmacists and nurses. Books and the internet exhibited a broader range of responses, with some skepticism. Trust in personal networks, such as friends and relatives, varied widely.

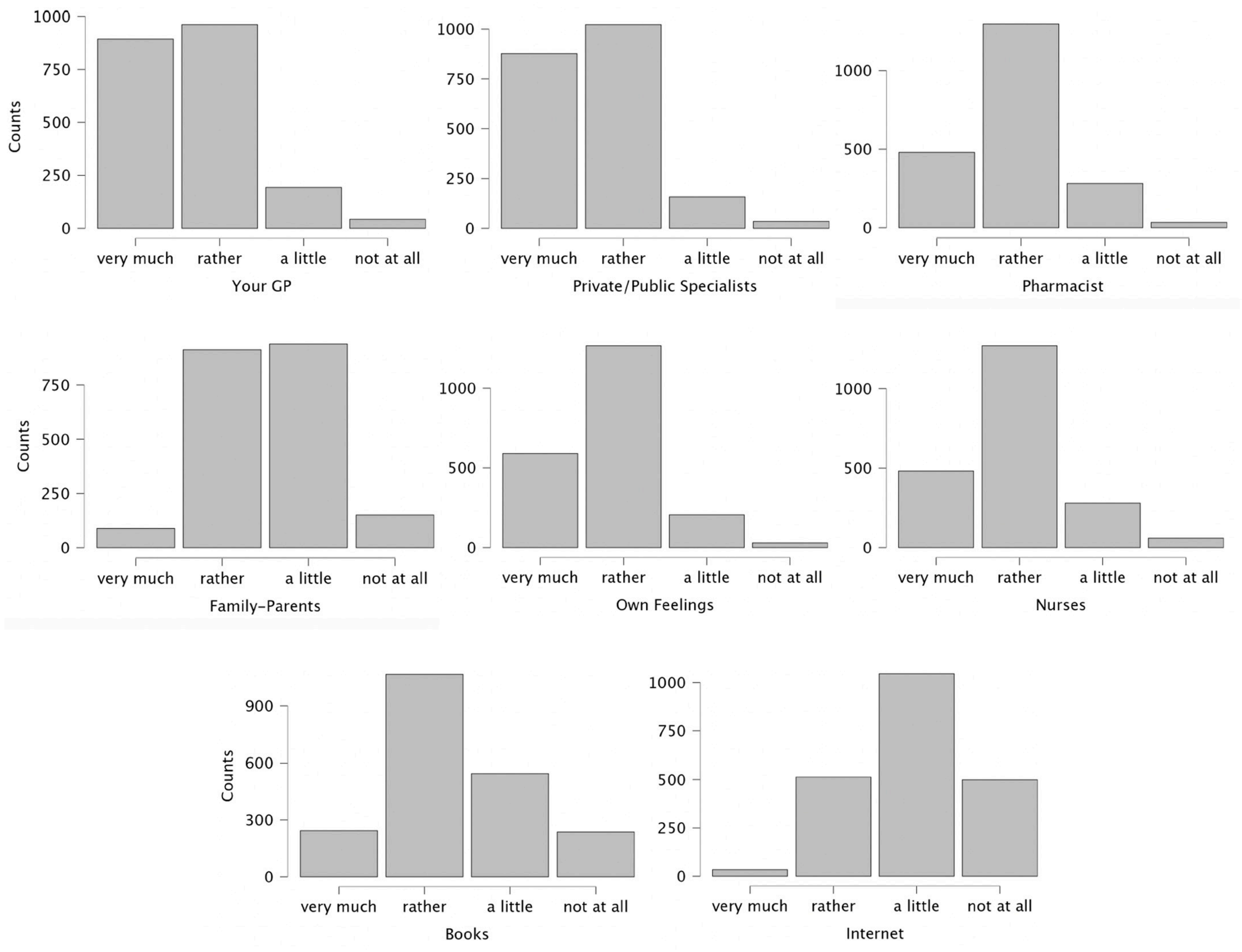

Figure S2: Distribution of trust ratings for various sources of health information (n = 2090).
